# Global priorities in HIA research: a new agenda for the next decade

**DOI:** 10.1186/s12889-025-21983-2

**Published:** 2025-02-26

**Authors:** Fiona Haigh, Liz Green, Katherine Hirono, Odile C. L. Mekel, Margaret Douglas

**Affiliations:** 1 International Centre for Future Health Systems, UNSW Sydney, Sydney, Australia; 2 Health Equity Research Development Unit, Sydney Local Health District, Sydney, Australia; 3 Policy and International Health, WHO Collaborating Centre Directorate, Public Health Wales, Cardiff, UK; 4 Department of International Health, Care and Public Health Research Institute – CAPHRI, Maastricht University, Maastricht, Netherlands; 5 RPS Consulting UK & Ireland, Edinburgh, UK; 6 NRW Centre for Health (LZG.NRW), Bochum, Germany; 7 Public Health Scotland, Edinburgh, UK; 8 University of Glasgow, Glasgow, UK; 9 Department of Health Policy and Management, Johns Hopkins Bloomberg School of Public Health, Baltimore, MD USA

**Keywords:** Effectiveness, Health equity, Health Impact Assessment (HIA), Health in all policies, Institutionalisation, Methodology, Participation, Research agenda

## Abstract

**Background:**

Health Impact Assessment (HIA) advances Health in All Policies by identifying impacts of proposed actions on health and equity and recommending changes to address these impacts. Since the Gothenburg Consensus Statement in 1999, HIA has been applied to policies, plans, programmes and projects in multiple sectors and settings across the world. Despite demonstrated effectiveness, its use across the world is inconsistent with few nations systematically using HIA. In a global context of increasing health inequities, pandemics, climate change, and economic crises, HIA can help integrate health and equity into decision making. There is a need for research to support the ongoing evolution and development of HIA. This paper presents a research agenda for the field of HIA.

**Methods:**

We used a mixed method approach utilising insights of approximately 280 participants through an international online survey and participatory workshops. We compared priority areas of research identified through the survey, workshops, and literature review to inform the development of a research agenda. The team drew on their own positioning and experience as HIA practitioners and researchers to shape this agenda.

**Results:**

We identified four research priorities: (1) Institutionalisation - Sustaining and institutionalising HIA in varying contexts and levels. (2) Influence - Identifying mechanisms and strategies that can be employed to effectively influence stakeholders and decision making. (3) Equity and Participation - Analysing the role of equity, justice, power and participation in HIA, and (4) Methodology - Improving HIA Methods to understand the complex relationships between proposed actions, health and health equity outcomes and identifying what to do. We developed research questions for each theme.

**Conclusions:**

The research agenda advocates for sustained research and practice to strengthen impact and address knowledge gaps in the field. Functioning as a roadmap for both researchers and funders, it aims to contribute to a healthier and more equitable world. Recognising the valuable role of HIA in addressing global health challenges, the agenda encourages researchers to investigate, develop, and advance the field of HIA.

**Supplementary Information:**

The online version contains supplementary material available at 10.1186/s12889-025-21983-2.

## Background

Health Impact Assessment (HIA) is a systematic process that uses scientific data, expert knowledge, and input from stakeholders to predict the potential public health and health equity impacts of initiatives. It also proposes measures to mitigate negative health effects and enhance positive ones. HIA has been applied to policies, plans, programmes and projects in multiple sectors in many settings across the world [1, 2] and its effectiveness in influencing policies has been highlighted by researchers and practitioners [3–6]. However, its use across the world is still variable with few nation states or regions using HIA systematically to maximise the potential of all policies and plans to improve health and address inequalities. Some nations have legislation that requires environmental health impact assessment but a formal requirement for HIA that encompasses assessment of social determinants of health and equity is rare [1, 7, 8].

HIA practice developed from experience of Environmental Impact Assessment (EIA) and Healthy Public Policy in the late 1990s [9]. Since then, it has emerged as a practical and robust public health approach through which to consider the impact of policies, plans and projects on the health and well-being of populations and any inequalities created by these. It is recognised as a beneficial tool to drive consideration of ‘Health in All Policies’ [10–12]. HIA has been embraced globally by the World Health Organisation (WHO), some governments and nation states but most research and practice has taken place in Europe, North America and Oceania [1, 12, 13].

A recent bibliographic analysis of published journal papers and research activities for HIAs since 2015 depicts a field of diverse interests and perspectives which have emerged as HIA has evolved [14]. They range from investigating health in EIA, transport and active travel HIAs, quantitative modelling, evaluation and effectiveness and social determinant and equity driven HIA case studies. The paper also identifies different paradigms which are operating in the field of HIA and as part of considering health in Environmental Impact Assessment (EIA). The analysis identified different perspectives on how HIA methodology is theorised and practiced. One world view to knowledge generation and evidence focuses on what is observable and measurable, leading to HIAs based on quantifiable impacts including environmental health determinants such as air quality, noise, emissions and these being the primary sources of evidence to create an understanding of the health impact of a proposal. A separate, alternative world view uses knowledge generated through both quantitative and qualitative evidence and data, including sociological evidence such as interviews and real-world experience to create a picture of health impacts. This can create conflict in the field or lead to fractionalisation in perspectives around the theory and practice of HIA [12, 15]. Methodologies and perspectives have taken advantage of legislative drivers such as EIA integration and events such as the COVID-19 pandemic to seize opportunities to include health and equity [16–18].

The Gothenburg Consensus Statement identified four key values for HIA: democracy, equity, sustainable development and ethical use of evidence [2]. These values were confirmed and expanded in the International Association for Impact Assessment (IAIA) published International Best Practice Principles for HIA, namely with Equity and Equality; Participation; Ethical use of evidence; Sustainability; and Comprehensive approach to health [19]. How these values are integrated into HIA guidance and practice varies [20]. Reducing inequities is a core principle and part of most HIA definitions. However, in practice HIAs often fail to adequately consider impacts on different population groups [21, 22]. In response, specific equity focussed approaches to HIA have been developed [22–24].

The COVID-19 pandemic has demonstrated the importance of social determinants of health and health equity, as many health and wellbeing impacts arose from the response to the unevenly distributed pandemic effects on health pathways [25, 26]. HIAs of pandemic responses have demonstrated its potential to identify and help address these impacts [16, 18, 27]. The climate emergency will bring even more wide-ranging effects on health that need to be addressed in an integrated way, which HIA could support [28, 29]. Post pandemic, it is timely to consider what is needed to support the future evolution and further development of HIA in different contexts and the research needed to support this development.

Despite growing knowledge and support for HIA, researchers and practitioners need to address remaining gaps. These include assessing predictive accuracy, increasing the number of case study examples in different sectors and settings and the institutionalisation of HIA [11]. Development in HIA research is needed to address gaps and advance the field.

## Methods - approach to developing the research agenda

**Fig. 1:**
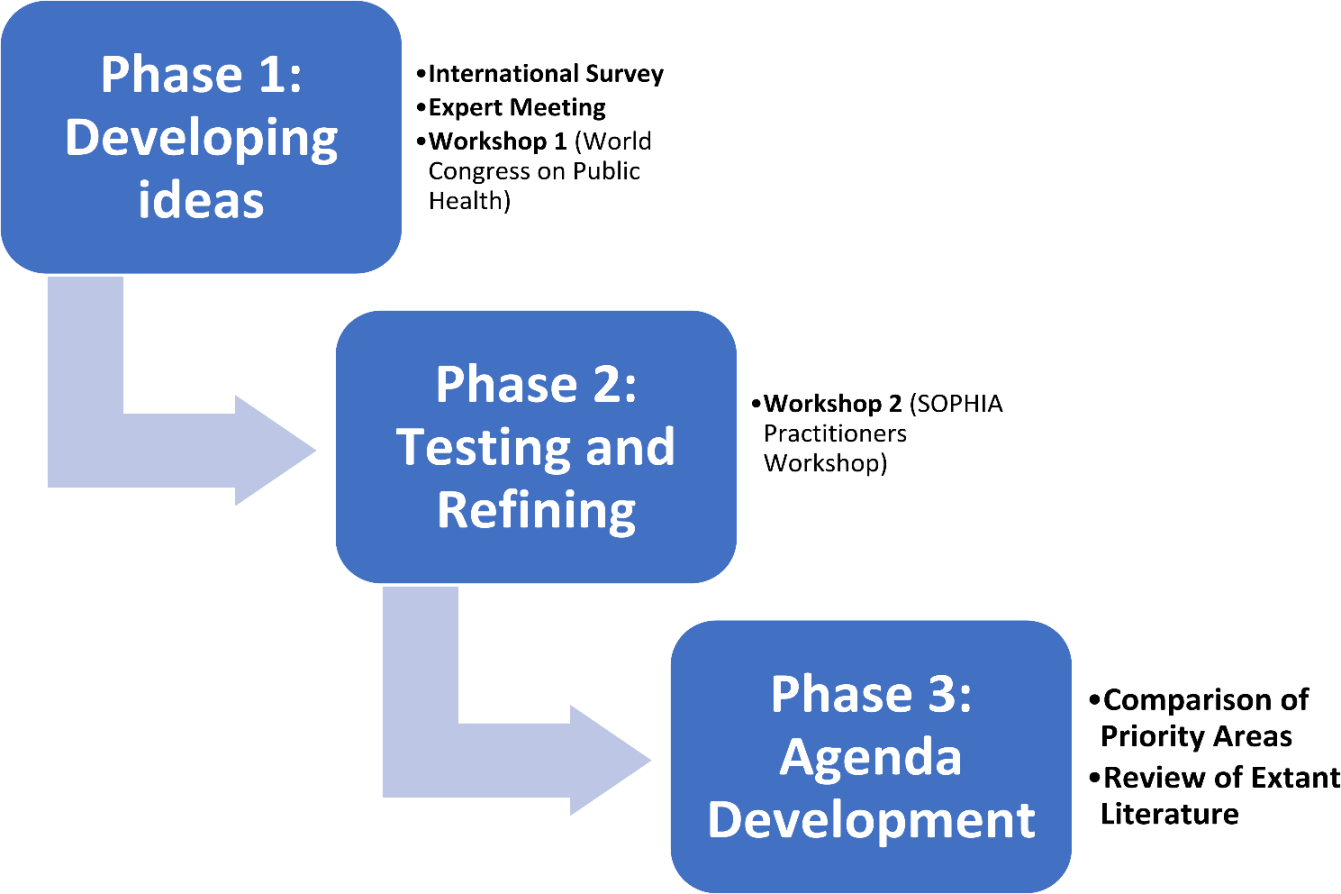
Research phases and methods diagram

To develop a research agenda for HIA, we followed several steps using a mixed method approach to data and evidence collection including a survey, and qualitative interactive workshops [30] (see Fig. 1).

## Data collection

### Phase 1: Developing ideas

Phase 1 involved an international survey of HIA practitioners. We developed a short anonymous self-administered online survey using the Qualtrics platform (see supplementary document 2). Ethical approval was granted by University of New South Wales Human Ethics Advisory Panel (HC220581). An invitation to complete the survey was circulated through existing email networks including the HIANET, the International Association for Impact Assessment HIA section, the European Public Health Association HIA Section, Society of Practitioners of HIA, and the International Union for Health Promotion and Education Global Working Group on HIA. The survey was open for 14 weeks between August and December 2022. Our inclusion criteria were: HIA practitioners, academics, educators or commissioners or other relevant HIA experience (self-identified). The survey asked the following: the participant’s current role, country currently based in, background in and experience of HIA, their views on important areas of research for HIA, the priority research questions and how to build and support HIA research, what they think the research needs are and how we could support the development of HIA research over the next 10 years.

The closed questions were descriptively analysed. Two members of the team (FH and MD) separately carried out thematic analysis of the open questions to identify key themes in relation to the questions;

- *What do you think the most important areas of research needed in the field of HIA in the next ten years?*
- *What do you think the most important unresolved research questions in HIA are?*
- *How can we build and support HIA research? What are important factors/ next steps/ approaches that we should be considering?*

The research team met to discuss these and resolve any difference in themes identified.

Our analysis of findings was also informed by a one-day meeting drawing on our experience as international HIA experts and leaders who have been involved in HIA development and advancement in different settings for more than two decades. We used preliminary findings from the survey and reflections on our experiences to inform a structured discussion on: the current opportunities for HIA and how to sustain HIA in a post pandemic context; differing practice and perspectives on HIA; potential methodological innovation; and research gaps and needs in the field.

#### Workshop 1

Approximately 60 participants at the World Congress on Public Health (WCPH) 2–6 May 2023 self-selected to attend a workshop titled ‘The future of Health Impact Assessment- Setting the Research Agenda’ [31]. During the 60-minute workshop, participants were presented with an overview of findings from the survey. We held small group discussions with workshop participants to: [1] discuss areas of research and gaps to address in the field of HIA over the next ten years [2], identify mechanisms to build HIA research capacity [3] prioritise research areas identified by the small groups. Individual participants wrote ideas on ‘post it’ notes that were then reviewed and discussed by all participants. Participants could also identify ideas they thought were priorities.

Two members of the research team (FH and MD) separately analysed the collated post it notes and identified key themes. A meeting was then held with the research team where any difference in theme identification between the reviewers was resolved by discussion.

### Phase 2: Testing and refining

#### Workshop 2

Two members of the team (KH and MD) presented the key themes to participants of the online SOPHIA Practitioners Workshop on 6th December 2023. Participants were invited to discuss further research questions for these themes in small online group discussions lasting 30 min. The research questions were recorded on a shared online document.

### Phase 3: Agenda development

The team then compared the priority areas of research identified by the survey and the two workshops. This was supplemented by reviewing extant literature particularly focussed on the role of HIA and state of the field papers (e.g. [1, 6, 12–14, 20]). The team developed these insights into an agenda, which draws on the research findings but also reflects our own positioning and experience as HIA practitioners and researchers.

## Results

### Participants

The varying stages of this work involved different participants at different times (see Table 1). Participants in the online survey were from international settings and reported a diversity of HIA practice, summarised below. For the in-person workshop held at the World Congress of Public Health, approximately 60 people attended. We did not collect any specific data on the workshop participants (e.g. experience, country of work). However, through examination of participants names and organisations (listed on their badges) and a short poll-taking activity at the start of the session, we determined that they were from a range of geographic areas including North America, Europe and Africa with a mix of levels of experience and knowledge of HIA. Fifty-three participants attended the SOPHIA practitioner workshop. Attendees of this workshop were practitioners of HIA and Health in All Policies, predominantly but not solely from the United States.

**Table 1:** Number of participants

Phase 1-Developing ideas	Phase 2 – Testing and refining
International Survey *n* = 166 Expert Meeting *n* = 5 Workshop 1 *n* ≈ 60	Workshop 2 *n* = 53
Total 231	Total 53

A total of 166 participants completed the online survey with number of responses to individual questions varying. A summary of the survey participant characteristics is provided in Supplementary data 2. Most survey participants were either academics (56%) and/or HIA practitioners (49%). There was a wide range of years of experience with most respondents having between six and twenty years of experience in HIA though 21% of participants had less than 2 years and 10% of participants had more than 20 years. Most participants reported working at either a university or other educational institution (47%) though over a quarter of respondents (26%) reported working for a government authority. Only 22% of participants were not involved in some type of HIA network. It should be noted that all the professional HIA networks we identified are headquartered in either North America or Europe, reflecting the communities of practice in those regions. Similarly, survey respondents were mainly located in Europe and the Americas with the UK and the US having the highest number of participants (22 and 16 persons respectively). However, 23% of respondents were from Africa or Asia, demonstrating a growing field of practice in those parts of the world.

In terms of the types of HIA practiced by the survey participants, 63% of respondents reported routinely conducting standalone HIA, whilst 40% reported conducting HIA as part of another form of impact assessment. Respondents also reported routinely conducting other forms of health impact assessment (e.g., Equity Focused HIA (19%), Mental Wellbeing Impact Assessment (13%). Respondents reported involvement in different types of governance for HIA [32] with the most respondents involved in decision-support HIAs (decision support 62%, community led 45%, advocacy 40%, mandated 33%). Lastly, 88% of participants reported routine consideration of social determinants of health while 68% reported consideration of environmental health determinants and 63% reported consideration of determinants of health equity. Please see additional file 1 for further detail.

In summary, the survey participant characteristics represent a largely academic community with a considerable number of years of experience. Participants were active in HIA networks representative of mostly North American or European communities of practice. Participants reported routinely conducting both standalone HIA and HIA as part of other impact assessment processes, though the majority of these were not mandated.

The research team also informed the agenda-setting process through our participation in internal workshops. The five team members are all experienced HIA practitioners, educators and researchers. 

FH is an academic working in the field of public health specialising in health equity. She has a disciplinary background in Law, Social Science and Public Health and has been actively involved in the HIA field since 2002. FH uses a critical realist approach to research with a focus on how to integrate health and health equity into planning, decision making and systems, the role of different types of knowledge in decision making, and the relationship between health and human rights. LG is a public health specialist working in the public sector in Wales. She has worked both in NHS public health practice and holds a PhD in HIA. Her interests include using HIA as a tool to implement Health in All Policies approaches and she is an expert on spatial planning and health and trade and health. She is Programme Director for HIA at Public Health Wales and provides advice, guidance and training on HIA and is Director of the Wales HIA Support Unit. She has been involved in developing and practicing HIA since 2004. KH is an HIA consultant within the private sector and an adjunct fellow in higher education. She has worked on conducting, teaching and developing capacity for HIA for over a decade in the US, Australia and the UK, and has contributed to over 50 HIAs throughout her career. As an academic researcher she has used qualitative, interpretive methods to examine how HIA can be a participatory process that affects health equity. She holds a PhD in global health policy and a Master of Public Health. OM is head of the Healthy Settings at the North Rhine-Westfalian Centre for Health (Landeszentrum Gesundheit Nordrhein-Westfalen, LZG.NRW) and vice-president of the European Public Health Association HIA Section. With over 20 years of experience in the field OM is an established expert in HIA and health risk assessment with a main interest in quantitative approaches to HIA and its integration into local and regional healthy public policies. MD is a public health physician working in the public sector in Scotland. She has worked both in NHS public health practice and in academia. Her interests include using a Health in All Policies approach, working closely with policymakers in economic, spatial planning, transport, housing and other policy areas. She is also involved in public health education and training. She has been involved in developing and practicing HIA since 1998, was a rapporteur at the Gothenburg consensus conference, has co-authored HIA guidance, resources, and academic papers and developed HIA training and teaching.

#### Themes

The key research themes that emerged from the data were: institutionalisation, effectiveness, methodology, participation and equity. Details on the types of research suggested by participants in relation to these themes is presented in Supplementary Data 2. A summary of the research areas proposed by the survey and workshop participants is presented in Table 2 and is described below. The research agenda is presented in a separate section after the discussion.

Both survey respondents and workshop participants identified a need for research on the framing and understanding of health in HIA, and whether these vary by geography, discipline or other characteristics.

Institutionalisation of HIA was a strong theme in both the survey and workshops, with multiple respondents asking for research on how to raise awareness among decisions makers, how to ‘embed’ or ‘systematise’ HIA and Health in All Policies, and whether it should be mandated. These discussion highlighted tensions between mandating HIA and the risks of ‘tick box’ exercises. Some survey respondents placed these questions in a specific country or context, with others suggesting a need to map variations in practice or study how to adapt practice to different, especially low income and rural, contexts. Survey respondents also suggested research on integrating health into other assessments. Workshop 2 participants added suggestions for research on how differences in context might affect institutionalisation, including case study evaluations in different contexts. They raised a question about how to encourage private sector organisations to do HIA and comply with recommendations, and the interaction with Quality Assurance processes and requirements. They also suggested research to understand the capacity needed for HIA and how this compares with available capacity.

Research on the effectiveness of HIA was raised in the workshops and was a strong theme in the survey, with multiple respondents identifying a need for research on whether HIAs influenced changes to decision-making, and whether these changes led to improved health and reduced health inequalities. Linked to this, survey respondents also identified a need to compare outcomes of HIA with other processes and other impact assessments. Participants from workshop 2 also raised the question of how to define effectiveness – an HIA might be deemed effective for influencing a decision but ineffective in addressing community concerns or might influence some dimensions of health but not others. Different stakeholders may have varying perspectives on what constitutes an effective HIA and how this can be measured. Participants suggested greater consideration is needed for examining when and how an HIA is effective and for whom.

Both the survey and workshops raised some questions about the HIA process and methods, including which projects required HIA, and how to balance different aims – such as efficiency and participation – in an HIA. Survey respondents also raised many more specific questions about HIA methodology. These included the use and value of quantitative data tools including digital data, how to estimate and communicate uncertainty, monetisation of impacts and methods for specific outcomes such as mental health. Participants in both the survey and workshops called for greater consideration and research into interdisciplinary approaches. Workshop 2 participants raised questions about criteria for screening proposals for HIA, how to assess cumulative impacts, and HIA methods for monitoring after the proposal is implemented.

Many of the research questions suggested about methodology were about how to meet the HIA principles of participation and equity. Survey respondents and workshop 2 participants identified a need for research on effective community engagement including ways to involve communities facing inequities, how to bring together different types of knowledge (professional, expert, community) and the relationship between HIA and community empowerment. Equity was a strong theme in workshop 2, with participants in all groups raising questions about how to better include equity considerations in HIA. These included how to measure or assess how well an HIA has addressed equity, how to manage differences between the interests, values or priorities of different populations, and which HIA methods best align with equity principles.

Both survey respondents and workshop 1 participants also identified many topics that should be subjected to HIA including climate change, energy, digital technologies and health services. They also identified many areas for research to improve the evidence base for HIAs. Some of these were very specific – such as the effects of specific environmental exposures on children – whereas others were more general, for example calling for research on the impacts of social, economic and environmental conditions on mental and spiritual wellbeing.

Table 2 shows that both survey and workshop 1 participants identified similar mechanisms to build HIA research. These included funding, research collaborations, sharing of both HIAs and HIA research and some specific research approaches. Survey respondents suggested case studies, HIA evaluations and implementation science whereas workshop participants suggested storytelling and citizen science. Both also identified several mechanisms that were more directed towards building support for HIA practice such as raising awareness of HIA, seeking a legal mandate, HIA training and education (see Table 3). One survey respondent explicitly highlighted that support for research on HIA needed to be accompanied by support for HIA practice.

**Table 2:** Areas of research identified in survey responses and conference workshop

Research areas proposed	Survey (Online, August - December 2022)	Workshop 1 (World Congress Public Health, May 2023, Rome Italy)
Understanding of ‘health’ in HIA	✓	✓
Institutionalising HIA, barriers and facilitators	✓	✓
Adapting/making less costly in different contexts	✓	
Map variations in practice	✓	
Comparison with other assessments/approaches	✓	
Evaluation of HIA effectiveness or accuracy	✓	✓
Overall HIA process	✓	✓
Use of quantitative data and methods	✓ (multiple suggestions made)	
Other methods and evidence	✓	
Mitigation strategies	✓	
Equity	✓	
Participation	✓	
Topics for HIA	✓ (multiple topics identified)	✓ (multiple topics identified)
Evidence base – impacts of specific determinants	✓ (multiple topics identified)	✓

**Table 3:** Mechanisms proposed to build HIA research capacity, in survey responses and conference workshop

Mechanisms proposed to build research	Survey (Online, August - December 2022)	Workshop 1 (World Congress Public Health, May 2023, Rome Italy)
Funding	✓	✓
Collaborations	✓ (multiple collaboration models proposed)	✓ (multiple collaboration models proposed)
Raise awareness of HIA	✓	✓
Seek legal mandate for HIA	✓	✓
Training and education	✓	✓
Sharing HIAs and HIA research	✓	✓
Research approaches	✓ (suggestions included case studies, HIA evaluations and implementation science)	✓ (suggestions included Storytelling and citizen science)

## Discussion

In this section we discuss the findings of the research and then present the research agenda. Developing our HIA research agenda identified key areas for future research and ways to strengthen the field. To our knowledge, this research agenda is the first time that such an analysis of the future direction of research in health impact assessment has been carried out.

Overall, the findings from the various sources showed consistent emphasis on the need for further research on institutionalisation and effectiveness of HIA even with numerous studies [4, 5, 33] demonstrating HIA effectiveness. As workshop participants identified, there may be more research needed to examine different types of effectiveness for different stakeholders in different contexts. This suggests that different research and communications about HIA effectiveness are required.

There were some differences between responses from survey respondents and participants of workshop 1. Survey respondents showed a strong interest in HIA methodology, while workshop 1 participants did not prioritise this. Similarly, survey respondents identified equity and participation as areas where research could inform HIA practice, but this was not emphasised in workshop 1. This may be due to the different participant characteristics. As the survey respondents were mainly HIA practitioners, their priorities reflected their experience and more in-depth knowledge of the field. In contrast, workshop 1 participants had less experience with HIA and may have assumed that methodology was already well-established. Participants of workshop 2 were asked to discuss questions for pre-determined themes but were more similar to survey respondents in their suggestions, reflecting their greater experience of HIA. They showed a strong interest in equity and participation, which were reflected in the questions raised across all the themes discussed in the groups.

### Strengths and limitations

It is important to note that our definition of HIA may have differed from that of some participants, as indicated by the survey responses which suggested that some respondents were referring to quantitative impact assessment methods [34] rather than HIA as it is defined in the Gothenburg Consensus Statement [2]. Additionally, some responses indicated that certain respondents understood our question about how to build HIA research as how to build capacity to carry out HIAs. Many of the strategies proposed to strengthen research were aimed at increasing HIA capacity, with only one person explicitly recognising the need to support both practice and research. A further limitation is that our data can show the topics and questions that respondents suggested in our online survey and workshops, but provide limited insights into the reasons why participants suggested these questions and topics. Further qualitative methods such as in-depth interviews or focus groups could be used to explore these further and help to mobilise the research agenda further.

A strength of this project has been the extensive involvement of HIA and public health practitioners and researchers. However, our participants were self-selected and we may not have reached all relevant stakeholders and therefore a limitation is that some voices are, or could be, missing. Workshop 2 was predominantly practitioners from the US. Although workshop 1 was part of a global conference, it was held in Italy, and it is likely that European colleagues were more able to attend. Although we tried to engage broadly with people interested and involved in the practise and research of HIA, ultimately the agenda has been developed by white researchers located in institutions of the Global north (albeit one of them located in Australia). The research agenda that we have developed is our (the authorship teams) understanding of research priorities to progress the field. We have identified four streams of research and highlighted ways of working to address these areas.

### Research agenda

#### Research stream 1: Institutionalisation - Sustaining and institutionalising HIA within different contexts and levels (organisations, systems, regions, states)

While the field of HIA has grown and demonstrated its effectiveness in influencing policies, programs and projects, HIA remains underutilised. Adoption varies with levels of activity and support changing over time. Some suggest that mandating HIA is needed for it to be sustained despite changing political priorities, but others argue this runs the risk of it becoming a ‘tick box’ ineffective [12, 35].

Future research could clarify the purposes and expected outcomes of institutionalisation informing how to transition HIA from one-off events to a sustainably routinely embedded process. Questions include how to establish sustainable funding models and supportive environments; understanding the relationship between HIA and other governance initiatives; balancing statutory but meaningful implementation; and addressing potential barriers in different contexts. Related to institutionalisation is the question of how to strengthen awareness and perception of HIA to support greater recognition and acceptance.

#### Research stream 2: Influence - Identifying the mechanisms and strategies that can be employed to effectively influence stakeholders and decision making to promote and encourage better health outcomes and achieve health equity

There is already substantial research demonstrating HIA effectiveness in influencing decision [3, 5, 36, 37]. The next research phase involves broadening the focus from determining the efficacy of HIAs to exploring ***how*** HIA can be positioned and utilized to maximise its influence.

Strengthening HIA requires research that considers the political and policy contexts where health and health equity are often not prioritised as significant outcomes, tackling issues and concepts such as the role of power and knowledge. The development of more effective decision support requires moving beyond simplistic rational linear understanding of decision making to considering complex knowledge systems with diverse actors, institutions and ideas in real-world contexts.

There is a need to develop theories for understanding and explaining dimensions of effectiveness and how HIA can be effective. Research will need to draw upon a broad range of disciplines and theories, including political and social sciences, considering the interplay between individual actions (agency) and the broader social systems or frameworks (structure) in which those actions occur.

#### Research theme 3: Equity and Participation- Analysing the role of equity, justice, power and participation in HIA

HIA is underpinned by a core set of principles including participation and equity but perceptions of the role of equity and participation in HIA vary significantly. Within the field, there is ongoing tension about the role of HIA as a supposedly neutral technical tool for decision support and influencing decision-making processes to promote health and social justice [38].

In this research theme, we identify two main areas of study. Firstly, research that examines the role of HIA in relation to principles of health equity, social justice, human rights, and democracy (as initially outlined in the Gothenburg consensus paper). Secondly, advancing ways to conceptualise, identify, and address health equity and other related impacts such as human rights within HIA frameworks. This requires a shift from focusing predominately on identifying differentially affected population groups and health impacts to considering the causes of the unequal distribution of the determinants of health such as drivers of poverty and in-built bias in systems and settings.

#### Research stream 4: Methodology - Strengthening HIA Methodology to meet the challenge of understanding the complex relationships between proposed actions, health and health equity outcomes and identifying what to do

There is continued need to carry out research focused on methodological development. HIA practice is evolving with HIA increasingly being used to inform responses to large scale changes such as pandemics, climate change, and international trade agreements [12, 16, 39–41]. This signals a change in scope of HIAs to move beyond single actions or interventions to areas that incorporate multiple actions and multiple complex impact pathways (e.g. pandemic responses, multiple actions driving climate change). Research can strengthen HIA’s capacity to identify the complex relationships between proposed actions, health and health equity outcomes and also identify potential actions – to work out what to do in response to these complex challenges.

While HIAs draw on findings from existing research that confirm relationships between health determinants and health impacts, the research often does not delve into potential causal explanations for those relationships. Findings are described as correlations between changes in determinants of health and potential health and health equity impacts (if x then y). However, without understanding how impacts happen (which generative mechanisms were involved), it is difficult to develop recommendations to change those impacts.

Research needs to explicitly engage with epistemology (how we can know) to advance theory on the role of evidence and different research approaches in HIA. By engaging with epistemology, we can critically examine how different types of evidence are valued and utilised in HIA. This includes research investigating the role of paradigm perspectives and beliefs about the value of different types and sources of knowledge. This stream of research should look both inwards (how different types and sources of knowledge are incorporated into HIA processes and how HIA can enable or hinder the participation of diverse stakeholders and knowledge in HIA) and outwards (investigating the role of cultural, political and institutional structures, beliefs, and practices that influence how evidence is understood and valued).

This will require drawing on methodological developments in different disciplines. For example, systems approaches, multi-level modelling, ways of integrating different types of knowledge such as group model building, and political and critical approaches to understanding and explaining policy and decision making. Approaches should consider various contexts and levels of available resources.

### Ways of working – making research happen

A multi-faceted approach is needed to build and support HIA research. HIA networks and organisations need to advocate for the inclusion of HIA within funding streams. In addition to HIA specific research, opportunities should be identified to embed HIA research within broader research agendas such as research on Health in All Policies, other forms of impact assessment, or priority areas affecting health such as climate change. Participants also emphasised the importance of strengthening the profile of HIA research through pro-active dissemination using existing networks to share examples of research. Including HIA courses or integrating HIA into existing courses within educational programs (e.g. public health, built environment, planning economic, science and technology, political and social science, medicine) could increase interest in HIA research and support students to engage in research for example through Masters and PhD programs.

Our research agenda will require transdisciplinary research approaches that move beyond silos of disciplines working in parallel (or serially), integrating different types of knowledge along with theory-informed approaches that unpack what works, for whom, in what circumstances, and how [42]. Research on institutionalisation and effectiveness would benefit from and could build on, more international comparative explanatory case studies that investigate and compare different contexts and approaches [38, 43, 44].

As a field we also need to consider who identifies research questions and carries out research. HIA’s focus on change and influence means that HIA research is well suited to emancipatory research approaches that take as a starting point that society can be improved [45, 46] and participatory action research approaches that bring together researchers and participants in the research process as “co-constructors of knowledge” and directly informs change and transformation [47]. Given HIA’s role in approval processes in areas such as extractive industries (which often are working on behalf of large transnational corporations), decolonising the field is especially important. What and whose knowledge is included and what ways of knowing and being are valued in HIA needs to be explicitly considered and acted on [48].

Table 4 below summarises the research streams and questions.

**Table 4:** Global priorities in HIA research: research streams and questions

**Research stream 1: Institutionalisation - Sustaining and institutionalising HIA within different contexts and levels (organisations, systems, regions, states)**	
1. How can HIA be institutionally embedded to ensure sustained influence on decision-making processes? a. What are the main ways to create conditions to institutionalise and operationalise HIA? b. What capacity and resources are needed are needed at different levels (human, technical, financial) to institutionalise HIA? c. In what ways can we build decision-makers’ and other stakeholders’ understanding of the value of HIA and how to utilise findings in policy and project development? 2. What can we learn about institutionalisation from the evolution of, and global trends in, HIA and other impact assessments and similar frameworks such as Social Return on Investment and HiAP? 3. What are ways of ensuring that HIAs carried out within mandatory processes reflect the principles of HIA a. What are the benefits and trade-offs of mandating HIA? b. What mechanisms or approaches can realise the benefits of mandated HIA while maintaining quality and effectiveness?	
**Research stream 2: Influence - Identifying the mechanisms and strategies that can be employed to effectively influence stakeholders and decision making to promote and encourage better health outcomes and achieve health equity.**	
4. What enables the consideration of HIA findings and recommendations in decision making processes? a. What mechanisms can be employed in HIA processes to influence decision making and planning processes (governance structures) to address potential impacts on health and health equity (how, for whom, in what circumstances, and why)? b. How can these mechanisms be implemented in different contexts and with varying resources? 5. How can HIA be utilised to influence decision making to support health and health equity in the context of dominant ideas and drivers that often create and sustain health inequities? a. How can communication and framing of HIA results take account of and influence dominant ideas that contribute to poor health and help to maintain and create health inequities? b. How can HIA processes and methods tactically and strategically challenge and reshape dominant ideas and discourses that contribute to poor health and health inequities? 6. How can HIA effectiveness be conceptualised and understood beyond its direct influence on individual proposals? a. What are ways of conceptualising effectiveness and influence in HIA? b. How can we understand HIA’s role beyond influencing individual decisions to organisational and system level change?	
**Research theme 3: Equity and Participation- Analysing the role of equity**,** justice**,** power and participation in HIA**	
7. In what ways can the principles of equity, human rights, and social justice be integrated into both HIA process and findings? a. How and to what extent do quantitative and qualitative data collection practices in Health Impact Assessment align with equity principles and practices? b. How can capacity be built to enhance the understanding and application of HIA principles among diverse stakeholders? c. What are ways to strengthen HIA process and methods to consider and assess the causes of the unequal distribution of determinants of health? 8. How can HIA be utilised to empower communities? a. What methods and processes best incorporate community priorities and perspectives and obtain community support and input in each step of the HIA process? b. What approaches for stakeholder and community participation within HIA can affect various forms of power? 9. How can HIA enhance accountability in decision-making, specifically concerning social justice, human rights and sustainability considerations? a. What are ways to assess the effectiveness of HIA in promoting equity, social justice, and human rights in decision-making? b. How can the impact of HIA on decision-making accountability be monitored and evaluated over time?	
**Research stream 4: Methodology - Strengthening HIA Methodology to meet the challenge of understanding the complex relationships between proposed actions**,** health and health equity outcomes and identifying what to do.**	
10. What forms of knowledge, research and evidence does HIA require to understand complex relationships between proposed actions, health and health equity outcomes? a. How can we develop and strengthen HIA methodologies to address different populations, determinants, and risk factors, considering: • cumulative, synergistic, and intersectional effects; • short, medium and long term effects; • trade-offs and opportunity costs? b. What forms of evidence and research are needed to assess different types of impacts across different settings? • What processes, research methods and evidence are needed to understand impacts on social, environmental and economic determinants at local, national and international levels? • What are approaches that can be utilised in low-income settings and low-resource environments? 11. How can HIA contribute to understanding and addressing global challenges? a. How can HIA methodology strategically assess and respond global challenges such as climate change and sustainability, conflict, international trade, cost of living crisis, and artificial intelligence. b. How can HIA methods and processes be strengthened to recognise and consider the impacts of racism, xenophobia and other forms of discrimination? 12. What criteria should be used to assess the quality of HIA and how can they be applied in different contexts? a. To what extent do Health Impact Assessment (HIA) methods and outcomes align with the best practice principles described in Gothenburg Consensus Statement and IAIA Best Practice Principles, and what criteria can be developed to systematically assess this alignment?	

## Conclusion

Using an online survey and two workshops, we found there was consistency in the areas that participants identified as priorities for future research on HIA and some explicit key themes. Our findings emphasise the ongoing need for research on the institutionalisation and effectiveness of HIA. Improving HIA methods is essential to understand the complex relationships between proposed actions, health, and health equity outcomes. Additionally, our agenda introduces newer, more innovative elements that push the field. This includes developing methods and procedures for integrating various forms of knowledge to address different populations, determinants, and contexts. This involves focusing on the causes of the uneven distribution of health determinants, such as power dynamics in decision-making, commercial determinants of health, and different forms of knowledge. We believe this will require research to advance integrating principles of equity, social justice, human rights, and democracy into HIA.

This research agenda aims to advance Health Impact Assessment by identifying research priorities for the next decade. It underscores HIA’s important role in enhancing healthy decision-making, maximising positive health outcomes, and addressing health inequities.

This agenda, developed in a global context marked by increasing health inequities, major crises such as pandemics, climate change and war, and the rise of misinformation, highlights the necessity of HIA in addressing global challenges and promoting health equity.

We need to strengthen the field of HIA through continued research and practice to identify how we can mitigate adverse health effects and optimise beneficial impacts of policies, programmes and projects ultimately fostering a healthier and more equitable world.

Whilst the research agenda has been formulated based on the research gaps and priorities identified through expert workshops, surveys, and international workshops, it ultimately reflects our perspective. The agenda is not intended to be comprehensive or suggest that research in other areas is not needed or valuable. Recognising the need for ongoing research and practice to strengthen the impact of HIA, we present this research agenda as a roadmap to guide future endeavours. It serves as an agenda, a set of research questions, and a call to action for researchers, including ourselves, to grow, develop and push the field.

## Electronic supplementary material

Below is the link to the electronic supplementary material.

Supplementary Material 1

Supplementary Material 2

## Data Availability

The datasets generated by the survey research during the current study are available from the corresponding author on reasonable request.

## Abbreviations

HIA: Health Impact Assessment
EIA: Environmental Impact Assessment
WHO: World Health Organisation
SOPHIA: Society of Practitioners of Health Impact Assessment
HIANET: HIA email discussion group hosted by JiscMail academic mailing list service
