# Supplementary material for: Global priorities in HIA research: a new agenda for the next decade
Source: BMC Public Health. 2025 Feb 26;25:791. doi: 10.1186/s12889-025-21983-2 (PMC11866836; doi:10.1186/s12889-025-21983-2)
Supplement: Supplementary file 1 — Supplementary Material 1 [file 12889_2025_21983_MOESM1_ESM.pdf]

Block 1

A Research Agenda for HIA

Since the Gothenburg Consensus Statement in 1999, HIA has been applied to policies, plans and projects in multiple sectors in many settings across the world. Researchers have demonstrated its effectiveness in influencing policies. However, its use is still variable globally with few jurisdictions using HIA systematically to maximise the potential of all policies and plans to improve health.

A small group of HIA academics and practitioners are working on a Research Agenda for HIA for the next decade. We would be very interested in your suggestions for priority research questions for the development of HIA.

Please read through the attached (click below) Online Participant Information Statement and then if you want to participate click on the 'I agree to participate' button below if you wish to complete the survey)

Participant Information Statement

I agree to participate

- ○ Yes

About you

First we have a few questions to help us understand your background in relation to HIA

How would you describe yourself in relation to the field of HIA (you can select more than one option)

- ☐ HIA Practitioner
- ☐ Academic
- ☐ Educator/trainer
- ☐ Commissioner
- ☐ other relevant HIA experience

Other- please describe

How many years of experience do you have in HIA?

- ○ < 2
- ○ 3-5
- ○ 6-10
- ○ 11-20
- ○ more than 20 years

Which country are you currently based in?

What best describes your current employer

- ○ university or other educational institution
- ○ private (for profit) organization (e.g. consultancy)
- ○ non-profit organization
- ○ governmental authority
- ○ self-employed
- ○ not employed/other

Other- please describe

What HIA networks are you currently involved in?

- [ ] IAIA - HIA Section
- [ ] European Public Health Association - HIA Section
- [ ] The Society for the Practitioners of Health Impact Assessment
- [ ] International Union for Health Promotion and Education - Global Working Group on HIA
- [ ] HIANET
- [ ] No HIA networks
- [ ] Other

Other HIA Networks not listed above

What types of HIA are you routinely involved in? (can select more than one option)

- [ ] Stand alone HIA
- [ ] Health/HIA in EIA
- [ ] Health/HIA in SEA
- [ ] Health/HIA in SIA

- ☐ Mental Wellbeing Impact Assessment (MWIA)
- ☐ Equity Focussed HIA (EFHIA)/ Inequalities HIA
- ☐ Other

Other types of HIA not listed above

[ ]

What types of governance of HIA are you routinely involved in (can select more than one option). See https://www.sciencedirect.com/science/article/abs for definitions

- ☐ Community led
- ☐ Mandated (e.g. by legislation)
- ☐ Decision Support
- ☐ Advocacy

What types of health determinants do you routinely consider in HIAs (can select more than one option)

- ☐ Social determinants of health
- ☐ Environmental health determinants
- ☐ Determinants of health equity

HIA research

Please tell us what you think about HIA research needs

What do you think the most important areas of research needed in the field of HIA in the next ten years?

What do you think the most important unresolved research questions in HIA are?

How can we build and support HIA research?
What are important factors/ next steps/ approaches that we should be considering?

Is there anything else you would like to tell us?

Block 3

Powered by Qualtrics
