# Supplementary material for: Global priorities in HIA research: a new agenda for the next decade
Source: BMC Public Health. 2025 Feb 26;25:791. doi: 10.1186/s12889-025-21983-2 (PMC11866836; doi:10.1186/s12889-025-21983-2)
Supplement: Supplementary file 2 — Supplementary Material 2 [file 12889_2025_21983_MOESM2_ESM.docx]

# Supplementary data 1 Survey respondent characteristics

| **How would you describe yourself in relation to the field of HIA (you can select more than one option) (n 118)** | **Percentage** | **Count** |
| --- | --- | --- |
| Academic | 56% | 66 |
| Commissioner | 5% | 6 |
| Educator/trainer | 25% | 30 |
| HIA Practitioner | 49% | 58 |
| other relevant HIA experience | 18% | 20 |
|  |  |  |
| **How many years of experience do you have in HIA? (n118)** |  |  |
| less than 2 | 21% | 25 |
| 3-5 | 19% | 23 |
| 6-10 | 26% | 31 |
| 11-20 | 23% | 27 |
| more than 20 years | 10% | 12 |
|  |  |  |
| **What country are you based in? Geographic area (UN Geoscheme) (n 166)** |  |  |
| Africa | 5% | 9 |
| Americas | 14% | 24 |
| Asia | 10% | 16 |
| Europe | 34% | 57 |
| Oceania | 1% | 2 |
| Missing | 35% | 58 |
|  |  |  |
| **What best describes your current employer (n 118)** |  |  |
| university or other educational institution | 47% | 56 |
| private (for profit) organization (e.g. consultancy) | 16% | 19 |
| non-profit organization | 7% | 8 |
| governmental authority | 26% | 31 |
| self-employed | 2% | 2 |
| not employed/other | 2% | 2 |
|  |  |  |
| **Involvement in HIA networks (can select more than one option) (n 117)** |  |  |
| European Public Health Association - HIA Section | 36.8% | 43 |
| No HIA networks | 35.4% | 40 |
| The Society for the Practitioners of Health Impact Assessment | 20.5% | 24 |
| IAIA - HIA Section | 16.2% | 19 |
| HIANET | 10.6% | 12 |
| International Union for Health Promotion and Education - Global Working Group on HIA | 8.5% | 10 |
|  |  |  |
| **What types of HIA are you routinely involved in? (can select more than one option) (n 102)** |  |  |
| Stand alone HIA | 64% | 65 |
| Health/HIA in EIA | 41% | 42 |
| Health/HIA in SEA | 24% | 24 |
| Equity Focussed HIA (EFHIA)/ Inequalities HIA | 19% | 19 |
| Mental Wellbeing Impact Assessment (MWIA) | 13% | 13 |
| Health/HIA in SIA | 10% | 10 |
| Other | 10% | 10 |
|  |  |  |
| **Types of governance of HIA routinely involved in (more than one response allowed) (n 97)** |  |  |
| Decision Support | 62% | 60 |
| Community led | 45% | 44 |
| Advocacy | 40% | 39 |
| Mandated (e.g. by legislation) | 33% | 32 |
|  |  |  |
| **Types of health determinants routinely consider in HIA (more than one response allowed) (n110)** |  |  |
| Social determinants of health | 88% | 97 |
| Environmental health determinants | 68% | 75 |
| Determinants of health equity | 63% | 69 |

# Supplementary data 2 SUMMARY OF Research Priorities identified by SURVEY AND WORKSHOP PARTICIPAnts

**Research priorities for the HIA Methodology**

**Research priorities for HIA Practice**

**Research priorities for HIA values**

**Research priorities for future HIA topics**
